# Pancreatic laceration and portal vein thrombosis in blunt trauma abdomen

**DOI:** 10.4103/0971-9261.43026

**Published:** 2008

**Authors:** Rajul Rastogi, Satish K. Bhargava, Shuchi Bhatt, Sandeep Goel, Sumeet Bhargava

**Affiliations:** Department of Radiology and Imaging, University College of Medical Sciences and Guru Teg Bahadur Hospital, Dilshad Garden, Delhi - 110 095, India

**Keywords:** Blunt trauma, laceration, thrombosis

## Abstract

Injuries to the pancreas by blunt trauma are uncommon. The association of pancreatic injury with acute portal vein thrombosis secondary to blunt trauma abdomen is furthermore rare. The early diagnosis of the pancreas with injury to the portal vein is challenging and difficult. These injuries are associated with high morbidity and mortality, particularly if the diagnosis is delayed. Accurate and early diagnosis is therefore imperative and computed tomography plays a key role in detection. We present a case of child with a rare combination of pancreatic laceration and acute portal vein thrombosis following a blunt trauma to the abdomen. With extensive literature search we found no such cases has been described previously.

## INTRODUCTION

Pancreatic injury occurs in less than 5% of the major abdominal injuries either as a result of penetrating or blunt trauma. Many blunt injuries to the pancreas including injuries to the main pancreatic duct are diagnosed late, thereby ending with a high morbidity and mortality.

Abdominal trauma is a rare and poorly documented cause of portal vein thrombosis. This diagnosis is made when all the other causes have been ruled out. Other causes include cirrhosis, tumors and inflammation of the abdomen, coagulation disorders and hematological diseases, including latent myeloproliferative syndrome.

We report a case in which the child suffered a blunt trauma to the abdomen and developed pancreatic laceration and acute portal vein thrombosis and underwent proper management due to the prompt diagnosis by preoperative CT.

## CASE HISTORY

**Figure 1:**
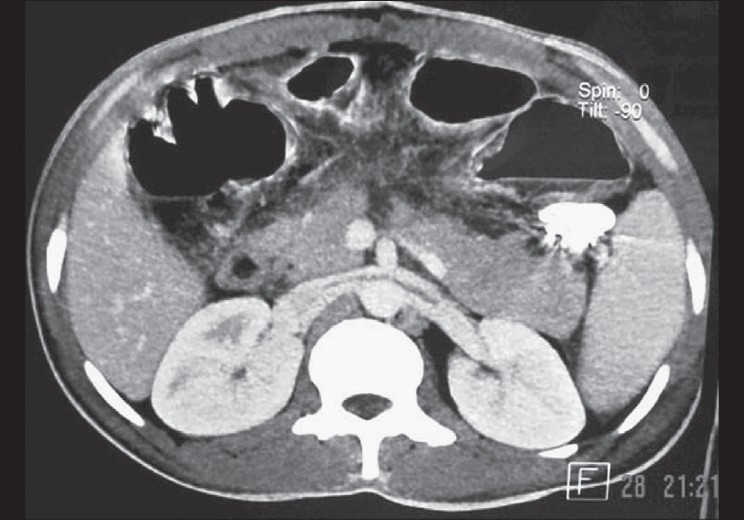
Axial CT image shows complete fracture through the neck of the pancreas, minimal peripancreatic fluid and soft tissue stranding in the peripancreatic fat

**Figure 2:**
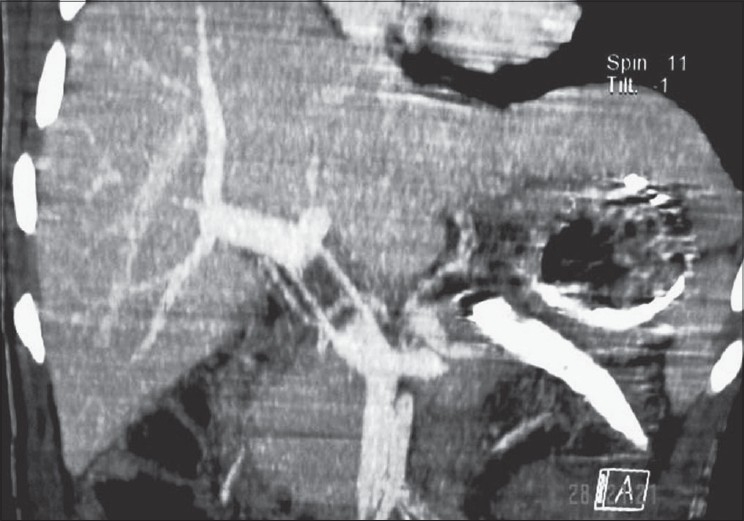
Coronal MPR CT image shows an acute main portal vein thrombus

An 11-year-old male child presented with acute epigastric pain following a fall on the handle of the bicycle. The clinical examination revealed signs of hypotension. His past and family history was unremarkable. Laboratory tests revealed increased ESR and increased serum amylase. There was no evidence of any myeloproliferative/hypercoagulable disorders. The X-ray chest and abdomen was unremarkable, and there was no free peritoneal air. Abdominal ultrasonography revealed doubtful laceration of pancreas in the neck region with minimal peripancreatic fluid. The rest of the abdomen appeared normal with no free peritoneal fluid. Emergency (within 6 h of the injury) contrast enhanced CT abdomen revealed a complete transection of the neck of the pancreas with peripancreatic fluid and soft tissue stranding suggestive of early pancreatitis [Figure 1]. The additional significant finding was a thrombus in the main portal vein [Figure 2] without any collateral vessel around the portal vein. The remaining part of the abdomen appeared normal.

Based on the clinicoradiologic and biochemical findings, the diagnosis of pancreatic laceration with ductal injury and acute portal vein thrombosis (PVT) was made. The diagnosis was confirmed at surgery and distal pancreatectomy with external drainage was performed. The patient was kept on anticoagulant therapy for acute PVT with constant monitoring. The postoperative period and follow-up to a period of 3 months was uneventful with complete resolution of portal vein thrombus.

## DISCUSSION

Pancreatic injuries due to blunt trauma abdomen are relatively uncommon. In adults, they occur most commonly during vehicular accidents; however, in children, such injuries occur during bicycle accidents. Ultrasound detects focal or diffuse pancreatitis or pseudocyst, but generally it does not depict the pancreatic fracture.[1] CT is the most effective modality to diagnose pancreatic fracture.[2] The pancreatic fracture is observed as a fracture line passing across the long axis of the pancreas, generally observed in the neck of the pancreas.[3] Other signs include pancreatic or peripancreatic hematoma, periduodenal hematoma, retroperitoneal fluid; edema of the peripancreatic fat or around the superior mesenteric vessels or thickening of the anterior perirenal fascia.[4]

In most pancreatic injuries, the attention is focused on the main pancreatic duct injury.[5] The presence of retroperitoneal fluid collection suggests pancreatic duct rupture, which requires emergent ERP.[1] CT is inadequate in demonstrating pancreatic duct rupture, but ERCP is 100% sensitive.[2,6] However, ERCP requires stable patients. Injuries of pancreas remain unrecognized during laparotomy in 8% cases with disastrous consequences.[2] Hence, the preoperative detection is imperative. Complications include bleeding, pancreatic abscess, recurrent pancreatitis, fistula formation and pancreatic pseudocyst. The mortality rate varies from 3-40%. The decision to operate depends upon the general condition of the patient and the findings through imaging. Pancreatic resection is usually the most suitable treatment if the CT scan or ERCP show that the duct has been damaged or transected.[7]

Abdominal trauma is a rare and poorly documented cause of PVT. According to Beaufort *et al*, only 8 cases have been reported in the literature.[8] Such injuries are usually associated with severe crushing forces, and hence, they can coexist with injuries involving the pancreas and retroperitoneum. The commonest finding is thrombosis; however, tearing or rupture may occasionally occur with the formation of periportal hematoma.

Ultrasound of the abdomen in an acute trauma patient may, in fact, miss acute PVT as hyperacute thrombus is anechoic, which is similar to the vessel lumen. The important criterion for diagnosis is thus clinical suspicion and performing the Duplex Doppler, which may reveal obstruction or alteration in the color and flow pattern. Contrast-enhanced CT is, however, diagnostic in acute thrombosis as is observed in our case. If unobserved, acute PVT may progress to chronic PVT with portal cavernoma and collateral formation with consequent portal hypertension.[9,10] Acute PVT should be treated with heparin followed by oral anticoagulation.[10]

To summarize, in a case of suspected or evident pancreatic injury, it is very important to rule out acute PVT to decrease the morbidity and mortality. CT provides the single, most important, noninvasive and quick tool to detect the pancreatic and related vascular injuries, particulary PVT, thereby assisting in better management.

## References

[1] Shuman WP (1997). CT diagnosis of blunt abdominal trauma in adults. Radiology.

[2] Dodds WJ, Taylor AJ, Erickson SJ, Lawson TL (1990). Traumatic fracture of the pancreas: CT characteristics. J Comput Assist Tomog.

[3] Bigattini D, Boverie H, Dondelinger RF (1999). CT of blunt trauma of the pancreas in adults. Eur Radiol.

[4] Farell RY, Kridge JE, Bornman PC, Knottenbelt JD, Terblanche J (1996). Operative strategies in pancreatic trauma. Br J Surg.

[5] Takishima T, Hirata M, Kataoka Y, Asari Y, Sato K, Ohwada T (1999). Pancreatographic classification of pancreatic ductal injuries caused by blunt injury to the pancreas. J Trauma.

[6] Wong YC, Wang LJ, Lin BC, Chen CJ, Lim KE, Chen RJ (1997). CT grading of blunt pancreatic injuries: Predilection of ductal disruption and surgical correlation. J Comput Assist Tomog.

[7] Wilson RH, Moorehead RJ (1991). Current management of trauma to the pancreas. Br J Surg.

[8] Beaufort P, Perney P, Coste F, Masbou J, Le Bricquir Y, Blanc F (1996). Post-traumatic thrombosis of the portal vein. Presse Med.

[9] Gonzalez F, Condat B, Deltenre P, Mathurin P, Paris JC, Dharancy S (2006). Extensive portal vein thrombosis related to abdominal trauma. Gastroenterol Clin Biol.

[10] Sheen CL, Lamparelli H, Milne A, Green I, Ramage JK (2000). Clinical features, diagnosis and outcome of acute portal vein thrombosis. QJM.

